# Impact of MMP-9 Genetic Polymorphism and Concentration on the Development of Coronary Artery Disease in Ukrainian Population

**DOI:** 10.1155/2022/2067632

**Published:** 2022-04-11

**Authors:** Oksana S. Pogorielova, Viktoriia V. Korniienko, Yaroslav D. Chumachenko, Olha A. Obukhova, Igor Martsovenko, Viktoriia Yu. Harbuzova

**Affiliations:** ^1^Department of Internal Medicine with Center of Respiratory Medicine, Sumy State University, Sumy 40007, Ukraine; ^2^Biomedical Research Center, Sumy State University, Sumy 40018, Ukraine; ^3^Scientific Laboratory of Molecular Genetic Studies, Sumy State University, Sumy 40007, Ukraine; ^4^Department of Physiology and Pathophysiology with Medical Biology Course, Sumy State University, Sumy 40018, Ukraine; ^5^Municipal Non-Profit Enterprise of Sumy Regional Council “Sumy Regional Cardiological Clinic”, Sumy 40030, Ukraine

## Abstract

Coronary artery disease (CAD) is one of the leading causes of death in Europe. It is known that atherosclerosis is the primary risk factor of CAD development. MMP-9 is involved in all stages of atherosclerosis and thus may contribute to CAD emergence. To investigate the influence of MMP-9 on the (CAD) development 25 patients with intact coronary arteries (CA), 40 patients with acute coronary syndrome (ACS), and 63 patients with chronic coronary syndrome (CCS) were enrolled in the study. Real-time PCR was carried out for genotyping on the rs17567-polymorphic locus, and ELISA study was performed to measure the MMP-9 plasma concentration. It was found the lower risk of MI occurrence for AG-carriers (*P*_*a*_=0.023; OR_a_ = 0.299, 95% CI = 0.106–0.848) in Ukrainian population.

## 1. Introduction

Coronary artery disease (CAD) is one of the main causes of death in European countries. To illustrate the sharp contrast between Eastern and Western Europe, the authors of the third edition of statistics on cardiovascular morbidity and mortality in European countries cite the example of Ukraine, where mortality from CAD among men under age 65 is 14 times higher and among women of the same age, it is 25 times higher than in men and women in France [1].

Atherosclerosis is characterized by a complex multifactorial pathophysiology that is the major cause of CAD [2]. This process starts with the accumulation of lipids, smooth muscle cell (SMC) proliferation, cell apoptosis, necrosis, and fibrosis [3]. Matrix metalloproteinases (MMPs) form a family of zinc-dependent enzymes with proteolytic activity against connective tissue proteins such as collagen, proteoglycans, and elastin. MMPs plays a key role in all stages of atherosclerosis through vascular inflammation, endothelial dysfunction, SMC migration, proliferation, and migration of vascular smooth muscle cells (VSMCs), increase of intima-media thickness, vascular calcification, extracellular matrix degradation, and promotion of endothelial cell apoptosis. MMPs participate in oxidative modification, low density lipoprotein effect, plaque activation, and destabilization [4–6]. Hypoxia and inflammation in the lesion can induce plaque neovascularization [7]. These pathological microvessels are more prone to rupture [8]. Plaque rupture or erosion may induce thrombus formation, leading to myocardial infarction or ischemic stroke [9]. Ruptured plaques are characterized by a large lipid-rich core, a thin fibrous cap that contains few SMCs and many macrophages, angiogenesis, and inflammation. Macrophages, the primary cells of the atherosclerotic lesions, express MMPs as proteolytic enzymes that may influence rates of atherogenesis and the stability of atherosclerotic plaques [10, 11]. Among MMPs, MMP-9 plays a crucial role in the development of atherosclerosis.

MMP-9, also known as gelatinase B or 92 kDa type IV collagenase, is one of the important members of MMPs that may contribute to the breakdown of the extracellular matrix (ECM). MMP-9 is characterized by wide substrates, but it possesses specialized proteolytic activity against type IV collagen, a major component of the coronary artery basement membrane underlying the endothelium and surrounding each VSMC [12]. MMP-9 is secreted from macrophages in the fibrous cap and has been suggested to be involved in the remodeling processes associated with atherosclerosis and plaque rupture [13, 14]. MMP-9 not only degrades ECM but also conducts a connection between the cell surface and the matrix [12, 15]. MMP-9 expression increases after vascular injury [16, 17] and is particularly evident in inflammatory atherosclerotic lesions [18]. MMP-9 contributes to plaque vulnerability, and its high expression of MMP-9 has been associated with coronary plaque destabilization [19].

It is very important to find specific mechanisms of atherosclerosis progression, the impact of genetic and external factors which influence the development of CAD in different populations. Therefore, the aim of this study was to investigate the influence of MMP-9 concentration and functional rs17576 single nucleotide polymorphism (SNP) on the risk of CAD development in the Ukrainian population.

## 2. Materials and Methods

### 2.1. Subjects

In our study, we enrolled 128 patients after coronary angiography from February to October 2019, regardless of the clinical presentation of acute or chronic chest pain, in the “Sumy Regional Cardiological Clinic” (Sumy, Ukraine). All medical procedures were provided in accordance with the Declaration of Helsinki (1964) and each participant was required to provide a written informed consent. The study protocol was approved by the Ethics Committee of the Medical Institute of Sumy State University.

**Figure 1:**
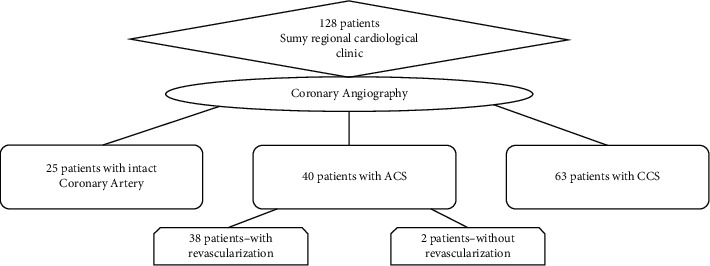
Scheme of patient recruiting and including to the groups.

After the coronary angiography, all patients were divided into three groups (Figure 1): group 1, 25 patients with angiographically normal (intact) coronary arteries; group 2, 40 patients with acute coronary syndrome (ACS); and group3, 63 patients with chronic coronary syndrome (CCS). The diagnosis of ACS and CCS and inclusion criteria of patients are provided according to the criteria described in the relevant ESC Guidelines [20–23]. The key exclusion criteria were as follows: any systemic connective tissue diseases, chronic obstructive pulmonary disease, bronchial asthma, and oncological pathology.

As a clinical routine, we recorded clinical data, including age, gender, weight, height, smoking habit, and the presence or absence of arterial hypertension (defined as systolic blood pressure >140 mmHg and/or diastolic blood pressure >90 mmHg and/or if subjects were receiving antihypertensive medication). All patients were examined using electrocardiogram (ECG) and echocardiography, and body mass index (BMI) was also calculated.

Troponin test was conducted for patients with ACS immediately after admission in hospital. Overnight fasting venous blood samples were collected from each subject for total cholesterol (TC), triglyceride (TG), low-density lipoprotein (LDL—cholesterol), high-density lipoprotein (HDL—cholesterol), AST, ALT, glucose, fibrinogen, erythrocytes, hemoglobin, leucocytes. The glomerular filtration rate was calculated with the QxMD Calculator (https://qxmd.com/calculate). Blood for detection of MMP-9 and rs 17567 MMp-9 polymorphism was collected under standardized conditions before coronary angiography.

38 patients with ACS received revascularization. Their medications included dual antiplatelet therapy (aspirin and P2Y12 receptor antagonist clopidogrel or ticagrelor), unfractionated heparin (only before revascularization), beta blockers (if they had no contraindications), rosuvastatin, and morphine. Nitroglycerine (iv) was added for some of those who had reccurent ischemia. ACE-inhibitors were prescribed to patients suffering from arterial hypertension or heart failure. 2 patients who did not receive revascularization have continued taking those medications in maintenance doses: aspirin and clopidogrel or ticagrelor, enoxaparin (5–7 days), beta-blockers, rosuvastatin, and ACE-inhibitors.

### 2.2. ELISA Study

Collected whole blood was kept in a tube without additives at room temperature for 30 min, followed by centrifugation at 3000 rpm at 4°C for 15 min. Serum was immediately transferred into a clean polypropylene tube and frozen at −80°C till further analysis, and recentrifuged after thawing before the assay. Levels of MMP-9 were assessed using a commercial ELISA immunoassay following the manufacturer's instructions (Platinum ELISA, Affymetrix eBioscience, BMS2016/2 MMP-9, Bender MedSystems GmbH). After reading each microwell's absorbance on a spectro-photometer, Thermo Scientific Multiskan FC (Thermo Fisher Scientific, Waltham, MA, USA) at 450 nm, the average absorbance values for each set of standards and samples in duplicate were calculated. To determine the concentration of circulating human MMP-9 (ng/ml) for each sample, a standard curve using computer software (Skanlt Software 4.1 for Microplate Readers) capable of generating a five parameter logistic (5-PL) curve-fit was prepared, taking into account the final dilution factor for prediluted and undiluted samples.

### 2.3. Genotyping

DNA extraction was performed from whole venous blood using the GeneJET Genomic DNA Purification Kit (Thermo Fisher Scientific, Lithuania). The genotyping for *MMP-9* rs17567-single nucleotide polymorphism was done by Real-time PCR using the 7500 Fast Real-time PCR System (Applied Biosystems, Foster City, USA) and Taq-Man Assays (TaqMan®SNP Assay C_11655953_10). The PCR conditions were as follows: denaturation step at 95°C for 45 s, treatment at 95°C for 15 s, and at 60°C for 30 s (45 cycles). The data obtained were processed with the 7500 Fast Real-time PCR Software.

### 2.4. Statistical Analysis

All statistical calculations were carried out using the Statistical Package for the Social Sciences software (SPSS, version 22.0, Chicago, IL, USA). The distribution normality of continuous variables was checked using the Kolmogorov–Smirnov test. Continuous parameters were presented as the mean ± standard deviation (SD) or median with interquartile range. Categorical variables were presented as absolute and percentage values. Normally distributed continuous parameters were compared using a two-tailed Student's *t*-test. The Mann–Whitney test was used to compare the continuous data with other types of distributions. All categorical variables, allele and genotype frequencies were compared using a chi-squared (*χ*2) test. The allele distribution in accordance with the Hardy–Weinberg equilibrium was detected by the calculator of Hardy–Weinberg equilibrium (https://wpcalc.com/en/equilibrium-hardy-weinberg/) for each group. The association analysis between MMP-9 rs17567-polymorphism as well as MMP-9 serum concentration and CAD development was performed using logistic regression. Genetic studies were carried out under dominant, recessive, overdominant, and additive models of inheritance. Further adjustments for age, sex, body mass index (BMI), the presence of arterial hypertension (AH), and smoking habit were incorporated to increase the reliability of the calculations. The value *P* < 0.05 was accepted as significant.

## 3. Results

Clinical and laboratory characteristics of various groups in the study are shown in Table 1. There were no statistically significant differences in gender distribution, as well as the number of patients with AH and smokers in the groups under comparison. Furthermore, the study also showed that various indices, namely, BMI, the levels of cholesterol, TG, LDL—cholesterol, HDL—cholesterol, glomerular filtration rate (GFR-EPI), erythrocytes, and hemoglobin are statistically insignificant both for ACS and CCS compared with the control group. In contrast, patients with ACS showed significant differences compared to the control group with respect to the following test parameters: glucose concentration (*P*=0.001), fibrinogen, ALT, AST, and MMP-9. Patients with CCS were significantly different from patients with intact coronary arteries with respect to age, concentration of glucose, concentration ща fibrinogen, and MMP-9.

The highest MMP-9 concentration was found in patients with ACS, the lowest in patients with intact coronary arteries, and patients with CCS had a middle range of MMP-9. Therefore, it indicated that a high concentration of MMP-9 is a risk factor for the progression of CAD and the development of its complication, ACS.

The results of association analysis between MMP-9 rs17576 and CAD development are shown in Table 2. There was a weak link with borderline significance between *MMP-9* concentration and ACS presence both before (*P*_*c*_=0.026; OR_c_ = 1.003, 95% CI = 1–1.006) and after the adjustment for covariates (*P*_*a*_=0.04; OR_a_ = 1.003, 95% CI = 1–1.006). In contrast, no significant association was detected for the CCS group (*P*_*a*_ > 0.05).

The results of the comparison of genotypes and alleles are shown in Table 3. There were no statistically significant differences in genotype and allele frequencies in the compared groups (*P* > 0.05). Genotypes are distributed in accordance with the Hardy–Weinberg equilibrium in each group (*P*_HWE_ > 0.05).

The association between *MMP-9* rs17576-polymorphic variant and the development of CAD was investigated using logistic regression (Table 4). There was no statistically significant link for both the ACS and CCS groups (*P*_*a*_ < 0.05).

The next step was to investigate the risk of myocardial infarction (MI) in patients with current CAD (47 among the 63 patients with CAD had previous MI in anamnesis). There was no statistically significant association between MMP-9 serum concentration and MI occurrence (Table 5).

A comparison of genotypes and alleles is indicated in Table 6. Statistically significant differences in genotype distribution (*P*=0.025) were found, while the allele distribution was similar (*P* > 0.05) among the compared groups.

It was found that AG-carriers had the lower risk of MI development in crude (*P*_*c*_=0.033; OR_c_ = 0.359, 95% CI = 0.14–0.922) and adjusted (*P*_*a*_=0.023; OR_a_ = 0.299, 95% CI = 0.106–0.848) overdominant model of inheritance (Table 7).

## 4. Discussion

The matrix-degrading activity of MMPs contributes to complications of atherosclerotic lesions. Vulnerable atherosclerotic plaques are responsible for life-threatening clinical endpoints: myocardial infarction and ischemic stroke [9, 24]. Plaque rupture is the most common cause of coronary thrombosis [25]. Thus, MMP-9 not only degrades ECM but may also contribute to plaque vulnerability [26].

Our study demonstrated that the concentration of MMP-9 is the highest in patients with ACS (449.4 ng/ml (95% CI = 151.15–624.5)) compared with patients with CCS (354.35 ng/ml (95% CI = 149.98–575.58)) and patients with intact coronary arteries (67.97 ng/ml (95% CI = 34.88–303.6)). Many other studies have shown that MMP-9 can serve as a biomarker of vulnerable plaques [18, 27–30]. It was revealed that patients with plaque rupture and ACS (myocardial infarction versus unstable angina pectoris) had significantly higher levels of MMP-9 than patients who did not have plaque rupture. Kobayashi et al. demonstrated that MMP-9 levels are elevated earlier than high-sensitive troponin T and have a higher diagnostic value for early stage of ACS [27].

Hamed and Abdel Fattah reported that patients with ACS who had adverse cardiovascular events had a higher level of MMP-9 [16]. MMP-9 levels can also be used to predict ischemic stroke and cardiovascular death in patients with 50% and more carotid stenosis [31]. Blankenberg et al. demonstrated a strong association between baseline MMP-9 levels and future risk of cardiovascular death [32]. We found a weak borderline association between MMP-9 serum concentration and CAD development that needs to be investigated in further studies with extended groups of comparison.

In our studies there was no statistically significant association between MMP-9 serum concentration and MI occurrence among comparison groups of patients who had MI in the past or at present and without MI (*P*_*a*_ > 0.05). Thus, the concentration of MMP-9 is not a prognostic factor for the risk of acute MI in a patient with current CAD among the Ukrainian population. The role of MMP-9 concentration in the risk of development of MI in patients who have current CAD has not been confirmed. The same results were obtained by Eldrup et al. [31]. However, Blankenberg et al. showed that high levels of MMP-9 in patients with stable and unstable angina were directly correlated with a high risk of cardiovascular death [32].

The human *MMP-9* gene, which is located on chromosome 20q12.2–13.1, contains 13 exons and 12 introns. Common polymorphic variants in the promoter and exon sequences have been reported to be associated with CAD in recent years. Zhang et al. showed that there was a functional single-nucleotide polymorphism (SNP) rs3918242: this nucleotide variation from C to T gave rise to a two-fold increase in the promoter activity and the T allele carriers tended to have a higher risk of severe CAD [33]. Another common SNP—rs17576 (or R279Q) located at exon 6, is an A to G substitution that results in change of positively charged arginine (R) to uncharged glutamine (Q) in the catalytic domain of the MMP-9 enzyme. Such alteration may have an effect on the activity of the enzyme, although few functional data are available [33–35]. It was assumed that the SNP may presumably be involved in the binding of the enzyme to its substrate elastin. Further bioinformatics studies identified the spectrum of microRNAs (miRNAs) that bind to SNPs within Chromosome 20 in an allele-dependent manner. Researchers have indicated 41 SNP-specific miRNAs that target MMP-9 polymorphic loci most of which (95%) are concentrated in the coding exon. It was shown that the stringent pairing between G-allele (rs 17576 SNP) containing MMP-9 transcript and hsa-miR-3934-5p (*δ*score = 167 and *δ*MFE = −20.99 kcal/mol) means that miRNA preferentially binds to mRNA with SNP-allele. It can be assumed that further hsa-miR-3934-5p-dependent deadenylation and decapping of MMP-9 transcript with G-allele will decrease the stability of mRNA and inhibit the protein translation. Thus, the potential mechanism of the posttranscriptional regulation of MMP-9 expression is carried out by miRNAs in an SNP-specific manner [36].

There were 7 case-control studies with 5525 cases of CAD and 2497 controls (Asian and European) related to the MMP-9 (R279Q) SNP concerning the risk of CAD [37]. The pooled results indicated a negative, but not significant, association between *MMP-9* (R279Q) gene polymorphism and CAD risk under all genotype models for the overall population and subgroup analysis. According to these data, heterozygotes (47%) predominated among the representatives of the European population with ACS, while homozygotes by AA genotype amounted to 42.5% in this group [14]. Homozygotes by AA genotype accounted for the majority (46.9–48.1%) among Asians with ACS, and heterozygotes were 39–43.9% [38]. The data are contradictory for the European population of patients with stable CAD: carriers of the AG genotype predominated (47.4%) among Norwegians, but in the Italian population the AA genotype is the most frequent (44.3%) [39, 40]. There are conflicting data on the distribution of genotypes by rs17567 *MMP-9* polymorphism among individuals with stable forms of CAD in the Asian population and among control groups of both populations [14, 35, 38–42]. In our study, we found that the minor allele frequency (MAF) in the ACS group was 31.4%, while in the CCS group it was 36.1%. That did not significantly differ from patients with intact coronary arteries (38.6%; *P* > 0.05).

The rs17567 MMP-9 (R279Q) polymorphism was evaluated in association with carotid artery stenosis [43, 44] and hypertension [34, 45]. The authors reported a strong association between the MMP9 279Q allele and the presence of atherosclerotic plaques in men. Although Blankenberg et al., revealed the association of the R279Q polymorphism with cardiovascular death and nonfatal MI (*P*=0.02) in the subgroup of patients with stable angina [32]. They showed that patients carrying the 279Q allele have a higher risk than patients homozygous for the 279R allele.

In current research, we did not find any significant link between rs17567 *MMP-9* and ACS or CCS development (*P*_*a*_ > 0.05).

Several studies have shown that R279Q SNP significantly increased the risk of MI in the premature CAD group [46, 47] as well as C-1562T polymorphism may increase susceptibility to myocardial ischemia [48]. When we compared the distribution of genotypes in groups of patients with and without MI, the statistically significant differences were found in genotypes (*P*=0.025) but not alleles (*P* > 0.05) distribution. Logistic regression analysis showed that AG-carriers had a lower risk of MI development in crude (*P*_*c*_=0.033; OR_c_ = 0.359; 95% CI = 0.14–0.922) and adjusted (*P*_*a*_=0.023; OR_a_ = 0.299; 95% CI = 0.106–0.848) overdominant models of inheritance. These results could be explained by the existence of ethnical features for different populations.

It should be noted that current research has several limitations. Further studies with extended groups are necessary to confirm the results of this study. Moreover, it would be interesting to measure the expression rates of *MMP-9* for each polymorphic variant.

## 5. Conclusions

In the present research, we have analyzed the link of MMP-9 serum concentration and *MMP-9* rs17567-polymorphic variant with CAD development among Ukrainians. We found a lower risk of MI occurrence for AG-carriers. However, further studies are required to confirm this observation.

## Figures and Tables

**Table 1:** Baseline characteristic of subjects.

Index	ACS, *n* = 40	CCS, *n* = 63	Intact, *n* = 25	*P*_1_	*P*_2_
Age, years^2^	58.35 ± 9.17	60.35 ± 7.95	55.4 ± 9.03	0.209^b^	0.013^b^
Gender, men/women	35/5	55/8	20/5	0.415	0.384
BMI, kg/m2^2^	28.69 ± 4.08	30.24 ± 4.97	31.18 ± 5.99	0.051^b^	0.452^b^
AH, *n* (%)	29 (72.5)	51 (81)	19 (76)	0.755	0.603
Smoker, *n* (%)	17 (42.5)	11 (17.5)	5 (20)	0.062	0.781
Cholesterol, mmol/L^2^	4.43 ± 1.15	4.44 ± 1.18	4.76 ± 1.67	0.365^b^	0.324^b^
Triglyceride, mmol/L	1.44 ± 0.75^1^	1 (0.84–1.29)^2^	1.24 ± 0.58^1^	0.295^b^	0.369^а^
HDL, mmol/L^2^	1.02 (0.89–1.14)^2^	1.09 ± 0.31^1^	1.07 (0.91–1.44)^2^	0.279^a^	0.439^b^
LDL, mmol/L	2.61 (2.2–3.38)	2.73 (2.07–3.44)	2.8 (1.99–3.36)	0.805^a^	0.628^а^
Glucose, mmol/L^1^	5.35 (4.6–6.9)	4.7 (4.2–5.78)	3.89 (3.53–4.58)	0.001^a^	0.01^а^
Fibrinogen, g/L^1^	2.2 (2–2.8)	3.7 (2.4–27.1)	3 (2.55–3.2)	0.003^a^	0.038^а^
GFR (ЕРІ), ml/min^2^	70.89 ± 21.87	74.41 ± 18.83	79.91 ± 27.8	0.242^b^	0.395^b^
АLТ, U/L^1^	99.05 (52.98–212.88)	26.35 (19.18–36.08)	25.35 (19.3–33.28)	<0.001^a^	0.811^а^
АSТ, U/L^1^	45.2 (26.78–66.58)	28 (22.6–35.9)	22.25 (19.88–27.73)	0.003^a^	0.168^а^
ММР-9,^1^	449.4 (151.15–624.5)	354.35 (149.98–575.58)	67.97 (34.88–303.6)	0.002^а^	0.004^а^
Erythrocytes, ×10^12^/L,^2^	4.52 ± 0.54	4.41 ± 0.51	4.46 ± 0.7	0.749^b^	0.733^b^
Hemoglobin, g/L^2^	148.34 ± 16.79	145.4 ± 14.84	147.11 ± 16.85	0.799^b^	0.679^b^

ACS: acute coronary syndrome; CCS: chronic coronary syndrome; intact: patients with intact coronary arteries; *n*: number of cases; BMI: body mass index; AH: arterial hypertension; HDL: high-density lipoprotein; LDL: low-density lipoprotein; GFR (ЕРІ): glomerular filtration rate (according to the chronic kidney disease epidemiology collaboration); АLТ: alanine aminotransferase; АSТ: aspartate aminotransferase. ^1^Data are given in the form of median and interquartile range. ^2^Data are given as mean and standard deviation. ^a^Comparison was performed using the Mann–Whitney criterion. ^b^Comparison was performed using Student's t-criterion. *P*_1_: *P* value for ACS and intact group comparison; *P*_2_: *P* value for CCS and intact group comparison.

**Table 2:** Analysis of the association between MMP-9 serum concentration and the development of CAD.

Predictor	*P*_*c*_	OR_c_ (95% CI)	*P*_*a*_	OR_a_ (95% CI)
ММР-9^a^	0.026	1.003 (1–1.006)	0.04	1.003 (1–1.006)
	0.058	1.003 (1–1.005)	0.06	1.003 (1–1.006)

CAD: coronary artery disease; *P*_*c*_: crude *P* value; *P*_*a*_: *P* value adjusted for age, sex, body mass index, the presence of hypertension, and smoking habits. ^a^Upper row represents the results for group with acute coronary syndrome and lower—for group with chronic coronary syndrome.

**Table 3:** Distribution of genotypes and alleles in comparison groups.

	ACS		CCS		Intact CA		*P*_1_ (*χ*^2^)	*P*_2_ (*χ*^2^)
	n	%	N	%	N	%		
Genotypes								
AA	18	51.4	21	38.9	8	36.4	0.472 (1.503)	0.946 (0.11)
AG	12	34.3	27	50	11	50		
GG	5	14.3	6	11.1	3	13.6		
Alleles								
A	48	68.6	69	63.9	27	61.4	0.43 (0.624)	0.77 (0.086)
G	22	31.4	39	36.1	17	38.6		
Hardy–Weinberg equilibrium								
*P*_HWE_ (*χ*^2^)	0.226 (1.464)		0.539 (0.378)		0.798 (0.065)		—	—

ACS: acute coronary syndrome; CCS: chronic coronary syndrome; intact: patients with intact coronary arteries; *n*: number of cases.

**Table 4:** Association analysis between *MMP-9* rs17576-single nucleotide polymorphism and CAD development.

Model^a^		*P*_*c*_	OR_c_ (95% CI)	*P*_*a*_	OR_a_ (95% CI)
Dominant		0.269	0.54 (0.181–1.609)	0.307	0.533 (0.159–1.781)
		0.837	0.898 (0.322–2.507)	0.611	0.751 (0.249–2.265)
Recessive		0.945	1.056 (0.226–4.936)	0.52	0.533 (0.078–3.628)
		0.758	0.792 (0.179–3.492)	0.787	0.793 (0.147–4.27)
Overdominant		0.242	0.522 (0.176–1.55)	0.498	0.647 (0.184–2.275)
		—	—	—	—
Additive	AG vs. AA	0.224	0.485 (0.151–1.558)	0.337	0.53 (0.145–1.938)
		0.903	0.935 (0.319–2.738)	0.67	0.781 (0.251–2.431)
	GG vs. AA	0.722	0.741 (0.141–3.88)	0.338	0.317 (0.03–3.319)
		0.74	0.762 (0.153–3.802)	0.949	0.936 (0.125–6.997)

CAD: coronary artery disease; *P*_*c*_: crude *P* value; *P*_*a*_: *P* value adjusted for age, sex, body mass index, the presence of hypertension, and smoking habits. ^a^Upper row represents the results for the group with acute coronary syndrome, and the lower one for group with chronic coronary syndrome.

**Table 5:** Association analysis between MMP-9 serum concentration and MI development among patients with CAD.

Predictor	*P*_*c*_	OR_c_ (95% CI)	*P*_*a*_	OR_a_ (95% CI)
ММР-9	0.169	1.001 (0.999–1.004)	0.237	1.001 (0.999–1.004)

MI: myocardial infarction; CAD: coronary artery disease; *P*_*c*_: crude *P* value; *P*_*a*_: *P* value adjusted for age, sex, body mass index, the presence of hypertension, and smoking habits.

**Table 6:** Distribution of genotypes and alleles in comparison groups.

	With MI		Without МІ		*P* (*χ*^2^)
	N	%	N	%	
Genotypes					
AA	29	46	10	38.5	0.025 (7.412)
AG	23	36.5	16	61.5	
GG	11	17.5	0	0	
Alleles					
A	81	64.3	36	69.2	0.527 (0.4)
G	45	35.7	16	30.8	

MI: myocardial infarction; *n*: number of cases.

**Table 7:** Association analysis between *MMP-9* rs17576-single nucleotide polymorphism and MI development among patients with CAD.

Model	*P*_*c*_	OR_c_ (95% CI)	*P*_*a*_	OR_a_ (95% CI)
Dominant	0.513	0.733 (0.288–1.862)	0.523	0.725 (0.271–1.942)
Overdominant	0.033	0.359 (0.14–0.922)	0.023	0.299 (0.106–0.848)
Additive	0.152	0.496 (0.19–1.296)	0.114	0.425 (0.147–1.23)

MI: myocardial infarction; CAD: coronary artery disease; *P*_*c*_: crude *P* value; *P*_*a*_: *P* value adjusted for age, sex, body mass index, the presence of hypertension, and smoking habits.

## Data Availability

The data used to support the findings of this study are available from the corresponding author upon request.
